# Illicit opioid use following changes in opioids prescribed for chronic non-cancer pain

**DOI:** 10.1371/journal.pone.0232538

**Published:** 2020-05-04

**Authors:** Phillip O. Coffin, Christopher Rowe, Natalie Oman, Katie Sinchek, Glenn-Milo Santos, Mark Faul, Rita Bagnulo, Deeqa Mohamed, Eric Vittinghoff

**Affiliations:** 1 San Francisco Department of Public Health, San Francisco, CA, United States of America; 2 University of California San Francisco, San Francisco, CA, United States of America; 3 University of California Berkeley, Berkeley, CA, United States of America; 4 Centers for Disease Control and Prevention, Atlanta, Georgia, United States of America; Medizinische Universitat Wien, AUSTRIA

## Abstract

**Background:**

After decades of increased opioid pain reliever prescribing, providers are rapidly reducing prescribing. We hypothesized that reduced access to prescribed opioid pain relievers among patients previously reliant upon opioid pain relievers would result in increased illicit opioid use.

**Methods and findings:**

We conducted a retrospective cohort study among 602 publicly insured primary care patients who had been prescribed opioids for chronic non-cancer pain for at least three consecutive months in San Francisco, recruited through convenience sampling. We conducted a historical reconstruction interview and medical chart abstraction focused on illicit substance use and opioid pain reliever prescriptions, respectively, from 2012 through the interview date in 2017–2018. We used a nested-cohort design, in which patients were classified, based on opioid pain reliever dose change, into a series of nested cohorts starting with each follow-up quarter. Using continuation-ratio models, we estimated associations between opioid prescription discontinuation or 30% increase or decrease in dose, relative to no change, and subsequent frequency of heroin and non-prescribed opioid pain reliever use, separately. Models controlled for demographics, clinical and behavioral characteristics, and past use of heroin or non-prescribed opioid pain relievers. A total of 56,372 and 56,484 participant-quarter observations were included from the 597 and 598 participants available for analyses of heroin and non-prescribed opioid pain reliever outcomes, respectively. Participants discontinued from prescribed opioids were more likely to use heroin (Adjusted Odds Ratio (AOR) = 1.57, 95% CI: 1.25–1.97) and non-prescribed opioid pain relievers (AOR = 1.75, 1.45–2.11) more frequently in subsequent quarters compared to participants with unchanged opioid prescriptions. Participants whose opioid pain reliever dose increased were more likely to use heroin more frequently (AOR = 1.67, 1.32–2.12). Results held throughout sensitivity analyses. The main limitations were the observational nature of results and limited generalizability beyond safety-net settings.

**Conclusions:**

Discontinuation of prescribed opioid pain relievers was associated with more frequent non-prescribed opioid pain reliever and heroin use; increased dose was also associated with more frequent heroin use. Clinicians should be aware of these risks in determining pain management approaches.

## Introduction

The United States opioid epidemic, initially driven by opioid pain relievers (OPRs), [1] has transitioned into a crisis driven by heroin and illicitly-manufactured fentanyl. [2, 3] First recognized by the Centers for Disease Control and Prevention (CDC) in 2007 [4], the crisis resulted in changes in prescribing policies and practices beginning in 2009. [5] Enforcement efforts focused largely on “pill mills,” [6] while clinical care measures have emphasized reduced prescribing through establishment of controlled substance monitoring programs (CSMPs), use of controlled substance agreements, opioid dose limits, and related strategies. [7]

In 2016, the CDC Guideline for Prescribing Opioids for Chronic Pain [8] included many recommendations for changes in opioid prescribing and management that have been implemented by health plans and clinic systems. The Guideline has since been linked to more rapid declines in opioid prescribing, high-dose OPR prescriptions (> = 90 morphine milligram equivalent [MME] per day), and concurrent OPR and benzodiazepine prescriptions. [9] However, reduced OPR prescribing has been associated, at least in some localities, with increased heroin overdose deaths. [10] Qualitative studies have also described associations between a shortage of prescription OPRs and increased illicit opioid use. [11, 12] Injection of illicit heroin is 3 times more risky for overdose than injection of OPRs [13, 14] and fentanyl 4 times more risky than heroin. [15] This potential shift from prescribed to illicit opioids could partially account for the observed increase in opioid overdose mortality, despite various efforts to implement changes in opioid prescribing and management by health systems and providers.

To improve our understanding during a national crisis of the potential association between reduced access to prescribed OPRs and illicit opioid use patterns, we designed a retrospective cohort study to capture and compare recent years of substance use and OPR prescription data.

## Methods

We conducted a retrospective cohort study of 602 publicly or uninsured patients in San Francisco who had been prescribed OPRs for chronic non-cancer pain (CNCP). Participants were seen once for a historical reconstruction interview focused on patterns of illicit opioid and other substance use; medical charts were then abstracted for data regarding opioid prescriptions, exposure to stewardship interventions, and other clinical measures. Study activities were completed at the San Francisco Department of Public Health from 2017–2018 and approved by the University of California San Francisco Institutional Review Board (IRB# 16–20458).

### Participants

**Fig 1:**
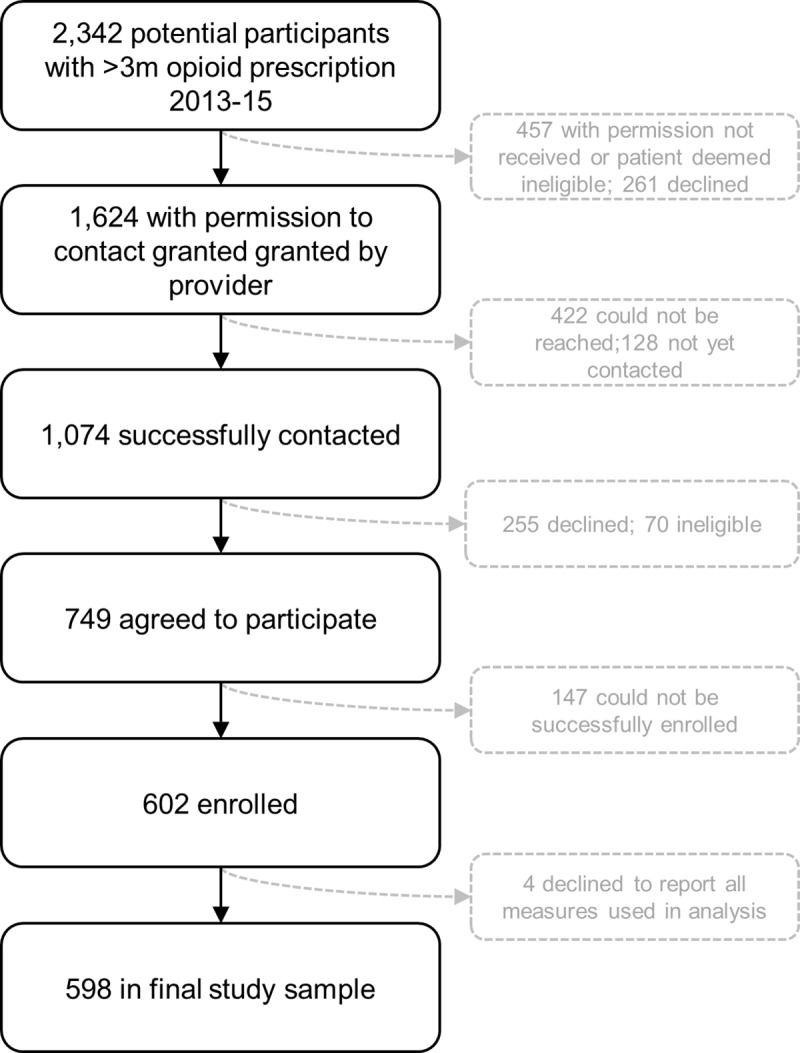
Recruitment flow chart.

Participants were ≥18 years of age, prescribed OPRs exclusively for CNCP for ≥3 months from 2013–2015, and able to communicate in English. Potential participants were identified through patient registries maintained by each clinic in the San Francisco Health Network (see **Fig 1**). Providers were contacted to obtain permission to contact patients. Interested patients were scheduled for an in-person visit, whereupon the historical reconstruction was completed. Participants were compensated $75 for their time. Following this visit, staff blinded to interview data conducted chart abstractions.

### Historical reconstruction procedures

At the study visit, the interviewer first collected demographic and lifetime substance use data. The historical reconstruction involved construction and visual display of a personal timeline from January 1, 2012, to the interview date (2017 or 2018) using major autobiographical landmarks (e.g., marriage, divorce, deaths, incarceration, housing transitions) as well as societal events (e.g., natural disasters, sporting outcomes, local news) relevant to each participant. The personal timeline was used to provide cues to facilitate more comprehensive and accurate recall of relevant events. [16] Measures using autobiographical landmark methods have been shown to be valid and reliable in assessing alcohol consumption. [17, 18] This visually displayed personal timeline served as a foundation for the reconstruction and dating of historical data collection.

To obtain historical data on heroin and non-prescribed OPR use, we modified a structured interview procedure from the Lifetime Drinking History interview and the Lifetime Drug Use Questionnaire, [19, 20] which construct substance use patterns over an entire lifetime in terms of different phases of use, defined by quantity and frequency. These phases are defined by the interviewee and begin with onset of regular substance use and end with current patterns of use, honing in on the dates when use patterns change and frequency of use within each distinct phase. These interview methods have been shown to have strong validity and reliability in retrospective assessment of substance use among multiple populations, including strong correlations with prospective measures, [21] highly significant test-retest correlations, [17–19, 22] and agreement between subject-collateral reports, [23] for up to 13 years in the past. [21, 24] In our modified approach, we limited the recall period to 2012 onward and defined periods as quarters (i.e., January-March, etc.). For example, if a participant reported use of heroin or non-prescribed OPRs, the interviewer would start by assessing use at the beginning of 2012, using the established autobiographical landmarks and societal events as reference points (discussing sensitive behaviors that occurred in the more distant past allowed for building of rapport and less stigma early in the interview [25]). The interviewer would then move forward in time using the visually displayed personal timeline and probe for changes in use. Different phases of use were added to the visual display and documented by the interviewer.

Each quarter included a code for frequency of heroin, non-prescribed OPRs, methamphetamine, cocaine, and alcohol use. Use frequency was coded as none, once, intermittent, less than weekly or weekly, multiple times per week, and daily or nearly daily, which we collapsed to none, once or intermittently (“intermittently”), less than weekly to multiple times per week (“weekly”), and daily or nearly daily (“daily”) to mitigate sparsity. The interviewer also documented episodes of using medications to treat opioid use disorder (OUD).

Interviews were conducted by trained staff after shadowing at least five interviews and being shadowed in an additional three interviews. Sessions were audio-recorded for review, to ensure comprehensive data collection and quality assurance.

### Chart abstraction procedures

Manual review of electronic medical charts of each patient was conducted after in-person visits by trained and blinded staff. The following data were abstracted by date from 2012 to study visit: opioid prescriptions (opioid type, dose, quantity per 30 days); exposure to opioid prescribing guideline interventions (naloxone prescription, controlled substance agreement, CSMP checks, yellow flag behaviors [such as ongoing illicit substance use] documented by the provider); and emergency department (ED) visits by opioid-relatedness. All abstracted charts were subsequently reviewed by the study physician to ensure accuracy and uniformity of coding.

### Longitudinal analysis measures

To be consistent with the timescale on which the outcomes were collected, the primary exposure of prescribed OPR dose change and other relevant longitudinal covariates were mapped to quarters.

Prescribed OPR dose change for each patient-quarter of follow-up was defined by comparing a patient’s daily dose in MME across all prescriptions on the first day of each quarter to their dose on the last day of each quarter. A dose increase was defined as any increase from zero MMEs or a relative increase of at least 30% from a non-zero starting dose; a decrease was defined as any relative decrease of at least 30%, excluding discontinuation; a discontinuation was defined as any decrease for which the patient ended the quarter on zero MMEs. The 30% threshold was used to capture clinically meaningful dose changes and aligns with the CDC’s most conservative taper recommendations. [26]

Longitudinal covariates were mapped to quarters and defined using their maximum values over lookback periods of one to four quarters prior to each dose change exposure quarter, using the lookback period duration for each covariate that maximized the log-likelihood in an unadjusted continuation-ratio regression model relating each covariate to each outcome. Prior alcohol use frequency was defined as maximum frequency reported during the lookback period; prior cocaine or methamphetamine use, opioid-related ED visits, yellow flag behaviors, controlled substance agreements, CSMP checks, naloxone prescription, and self-reported medications for OUD were defined as presence or absence of these at any time during the lookback period. Past values of outcomes were also operationalized as covariates in the same way and defined as the maximum frequency reported during the lookback period.

See **S1 Table** for detailed definitions and data sources for all analysis measures.

### Analysis design

Consistent estimation of the association between opioid dose change and subsequent use of heroin and non-prescribed OPRs was complicated by time-varying confounding, requiring specialized analysis methods. Specifically, providers may consider history of heroin and non-prescribed OPR use in prescribing OPRs, thus confounding our primary exposure by past values of the outcome. Similarly, other time-varying covariates (e.g., opioid-related ED visits) may both affect and be affected by the time-varying primary exposure. A repeated measures analysis adjusting for past outcomes and other covariates as time-varying confounders would not consistently estimate the overall effect of OPR dose changes, because the exposure effect would be partly mediated through covariates and past outcomes included as covariates in the analysis. [27]

To address this difficulty, we adopted a nested-cohorts approach, [28] in which effect estimates could be adjusted for baseline covariates only. In this set-up, follow-up for each participant began on January 1, 2012, or at entry into the San Francisco Health Network. The first year of follow-up was set aside for stable estimation of baseline dose and not used in defining nested cohorts. Subsequently, participants were included in multiple nested cohorts, one for each quarter, through the penultimate quarter. In each nested cohort, exposure and confounder history were fixed as of the cohort-defining baseline quarter. Follow-up for outcomes began in the next quarter and was censored at the first subsequent change in OPR dose or the final quarter of follow up for each participant. Outcomes occurring in the cohort-defining baseline quarter or the quarter of subsequent dose change were excluded to ensure that the exposure always preceded the outcome, and the outcome always preceded any subsequent dose changes. All nested cohorts were pooled for analysis.

### Statistical analysis

The main analysis used logistic continuation-ratio models (CRMs) to estimate the overall association between prescribed OPR dose change and each outcome, frequency of heroin and non-prescribed OPR use. CRMs estimated the log odds of reporting more than a particular frequency of use, given that the patient reported at least that frequency. The model thus implicitly made three conditional comparisons: (1) any use versus no use, in the overall sample; (2) weekly or daily versus intermittent use, in the subset with at least intermittent use; and (3) daily versus weekly use, in the subset with at least weekly use. The standard CRM assumed that the odds ratios relating each exposure to the outcome were constant across these three conditional comparisons. Because Wald tests showed that this assumption did not hold, we also present extended CRMs that allow the odds ratios to vary across the three conditional comparisons.

The models also controlled for potential confounders with either established or hypothesized relationships with both changes in prescribed OPR dose and use of heroin or non-prescribed OPRs. [29–35] Potential confounders that were essentially fixed (e.g., demographics) were included as baseline covariates that remained static over the study period whereas potential confounders that changed over time were included as lagged covariates that could change over the study period. Specifically, each model controlled for continuous patient age; race/ethnicity (non-Hispanic white, non-Hispanic black, Hispanic, non-Hispanic other/mixed race); gender (male, female, transgender or other); education (less than high school, high school graduate, some college less than Bachelor’s, Bachelor’s degree or higher); any heroin or non-prescribed OPR use prior to baseline (defined as the first quarter included in the analysis); mean OPR dose during the first year of follow-up (i.e., the year prior to baseline); and OPR dose change in the quarter prior to the cohort-defining quarter, the initial OPR dose that was used to define the dose change in the cohort-defining quarter. Each model also controlled for the following lagged covariates: alcohol use frequency (collapsed to none, intermittently, weekly, and daily); any use of either cocaine or methamphetamine; any opioid-related ED visits; any yellow flag behavior; any controlled substance agreement; any CSMP check; first naloxone prescription; and any self-reported medications for OUD treatment. The models also controlled for a lagged value of heroin or non-prescribed OPR use frequency as a single ordinal variable. Finally, we controlled for cohort-defining quarter and quarter of cohort follow-up; as with age, these continuous factors were modeled using restricted cubic splines if indicated by the Bayesian Information Criterion, and otherwise as linear.

Censoring by subsequent changes in opioid dose could be affected by outcomes and other confounders measured after each nested-cohort baseline. To avoid selection bias [36] without adjusting away indirect effects of OPR dose changes, we used time-dependent stabilized inverse probability of retention weights (sIPRWs). [28] To estimate the sIPRWs, we used two pooled logistic regression (PLR) models for quarters to subsequent dose change, the first including only covariates observed at nested-cohort baseline, and the second including time-varying post-baseline as well as the same baseline covariates. We then obtained the estimated probabilities of the participant remaining uncensored through each quarter, and finally, following standard methods, [37] calculated the sIPRWs as the ratio of the first and second probability estimates for each quarter.

All models used cluster-robust standard errors to account for repeated observations by participant. Analyses were conducted using Stata 15; CRMs were estimated using the user-written *gencrm* package. [38]

### Sensitivity analyses

To assess the sensitivity of results to the data-driven selection of covariate lookback periods, we generated models in which longitudinal covariate lookback periods were universally set to one or four quarters.

To assess the presence of interaction effects between prescribed OPR dose change and past illicit opioid use, we fit models that included interaction terms between prescribed OPR dose change and heroin use (for the heroin outcome model) or non-prescribed OPR use (for the non-prescribed OPR outcome model) prior to baseline. We also stratified each standard CRM model by heroin or non-prescribed OPR use prior to baseline.

In response to comments from a reviewer, we fit additional models that included any benzodiazepine prescription as a lagged covariate, with the optimal lookback period selected as described previously.

## Results

A total of 602 patients were enrolled; 598 were included in the final analysis for the heroin outcome and 597 for the non-prescribed OPR outcome. Four participants were excluded from both analyses because they declined to report alcohol, methamphetamine, or cocaine use during the historical reconstruction interview, and one participant was excluded from the non-prescribed OPR outcome analysis because they declined to report their use of non-prescribed OPRs. In total, 2,342 potentially eligible participants were identified from patient registries, of which provider permission to contact was obtained for 1,624; of these, 1,074 were contacted, 749 agreed to participate, and 602 were successfully enrolled (see **Fig 1**). Demographic characteristics and summary measures are presented in **Table 1**. Among the 2,342 potentially eligible participants identified from patient registries, there were no significant differences (*p* < 0.05 using chi-square and t-tests) between those who enrolled and those who did not with regard to gender (including only male and female patients, as these are the only defined genders in the electronic health record), race/ethnicity, or age.

**Table 1:** Participant baseline characteristics and longitudinal measures (n = 598).

Characteristic/Measure	n	(%)
Baseline Characteristics		
**Total Number of Participants**	598	
Age at baseline, median (IQR)*	52	(46–59)
**Race/Ethnicity**		
Non-Hispanic white	159	(26.6)
Non-Hispanic black	262	(43.8)
Hispanic	95	(15.9)
Non-Hispanic other/mixed race	82	(13.7)
**Gender**		
Male	345	(57.7)
Female	227	(38.0)
Transgender or other gender	26	(4.3)
**Education at end of follow-up (i.e., at time of study visit)**		
Less than high school	130	(21.7)
High school graduate	190	(31.8)
Some College, Associate's degree, or vocational training	224	(37.5)
Bachelor's degree or higher	54	(9.0)
Used illicit opioids prior to baseline*	300	(50.2)
Used heroin prior to baseline	224	(37.5)
Used non-prescribed OPRs prior to baseline	241	(40.3)
**Longitudinal Measures**		
**Number of quarters of follow-up, median (IQR)**	19	(18–21)
Mean opioid dose in year prior to baseline among participants prescribed opioids, mean (SD)*		
(# prescribed any opioids during year prior to baseline = 537 [90%])	182.0	(445.0)
**Mean opioid dose among participants prescribed opioids by year, *mean (SD)***		
2013 (n = 587; # prescribed any opioids during year = 550 [94%])	196.3	(407.4)
2014 (n = 598; # prescribed any opioids during year = 571 [95%])	173.1	(357.3)
2015 (n = 598; # prescribed any opioids during year = 532 [89%])	171.4	(338.9)
2016 (n = 598; # prescribed any opioids during year = 504 [84%])	167.7	(315.6)
2017 (n = 598; # prescribed any opioids during year = 467 [78%])	158.7	(295.9)
2018 (n = 459; # prescribed any opioids during year = 313 [68%])	163.2	(321.8)
**Experienced ≥1 opioid dose increase**	382	(63.9)
**Experienced ≥1 opioid dose decrease**	279	(46.7)
**Experienced ≥1 opioid dose discontinuation**	237	(39.6)
**Reported illicit opioid use during outcome follow-up**	181	(30.3)
Reported heroin use during follow-up	79	(13.2)
Reported non-prescribed OPR use during follow-up	151	(25.3)
**Initiated illicit opioid use during outcome follow-up**	38	(6.4)
Initiated heroin use during follow-up	9	(1.5)
Initiated non-prescribed OPR use during follow-up	31	(5.2)
**Reported alcohol use during follow-up**	381	(63.7)
**Reported cocaine or methamphetamine use during follow-up**	214	(35.8)
**Opioid-related emergency department visit during follow-up**	178	(29.8)
**Had controlled substance agreement during follow-up**	463	(77.4)
**Provider consulted CSMP during follow-up**	375	(62.7)
**Naloxone prescription during follow-up**	273	(45.7)
**Yellow flag behavior during follow-up**	354	(59.2)
**Medications for opioid use disorder treatment (self-reported) during follow-up**	96	(16.1)
**Overdose event (self-reported) during follow-up**	22	(3.7)

*Baseline is defined as the first quarter included in the analysis

The sample was older (median age 52), racially diverse (27% non-Hispanic white, 44% non-Hispanic black, 16% Hispanic, 14% non-Hispanic other/mixed race), and 9% had a Bachelor’s degree or higher. Half (50%) had used illicit opioids prior to baseline (38% heroin and 40% non-prescribed OPRs). The mean prescribed OPR dose was 196 MME (SD: 407) in 2013 and 163 (SD: 322) in 2018 among the 550 (94% of 587 participants under follow-up) and 313 (68% of 459 participants under follow-up) among participants prescribed OPRs during each year, respectively. A total of 38 participants (6%) initiated illicit opioid use for the first time during follow-up (9 [2%] heroin and 31 [5%] non-prescribed OPRs). The pooled nested-cohort dataset included a total of 56,484 and 56,372 participant-quarter observations for the heroin and non-prescribed OPR outcomes, respectively (median: 82, IQR: 53–126, quarter observations per participant for both outcomes).

**Fig 2:**
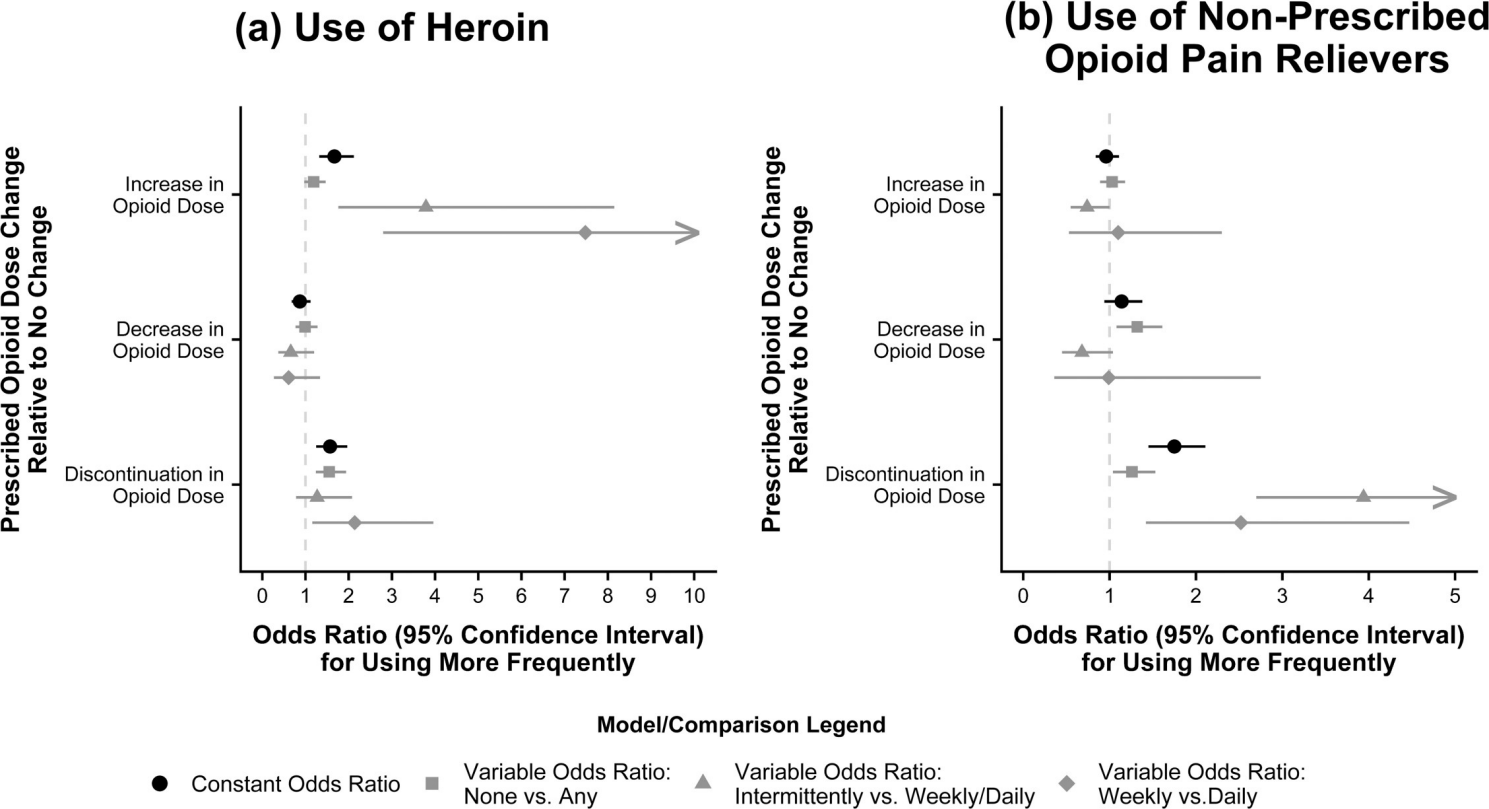
Use of (a) heroin and (b) non-prescribed opioid pain relievers, after change in prescribed opioid pain reliever dose.

In standard CRMs with constant odds ratios, participants discontinued from prescribed OPRs used heroin (AOR: 1.57, 95% CI: 1.25–1.97) and non-prescribed OPRs (AOR: 1.75, 1.45–2.11) more frequently in subsequent quarters than participants who had no change in their prescribed OPR dose, controlling for frequency of use prior to the dose change and other covariates (**Table 2** and **Fig 2**). In addition, participants whose OPR dose was increased used heroin (AOR: 1.67, 1.32–2.12) more frequently in subsequent quarters than participants who had no change in prescribed OPR dose.

**Table 2:** Multivariable continuation ratio regression assessing the association between changes in prescribed opioid dose and use frequency of heroin and non-prescribed opioid pain relievers.

		Continuation Ratio Model with Constant Odds Ratios		Continuation Ratio Model with Variable Odds Ratio					
				Any vs. None		Weekly/Daily vs. Intermittently		Daily vs. Weekly	
Outcome	Dose Change	OR	(95%CI)	OR	(95%CI)	OR	(95%CI)	OR	(95%CI)
More Frequent Heroin Use	No Change	Reference		Reference		Reference		Reference	
	Increase	1.67	(1.32–2.12)	1.19	(0.97–1.47)	3.79	(1.76–8.15)	7.48	(2.80–20.03)
	Decrease	0.87	(0.68–1.12)	0.99	(0.77–1.28)	0.66	(0.37–1.20)	0.61	(0.27–1.34)
	Discontinued	1.57	(1.25–1.97)	1.55	(1.24–1.94)	1.27	(0.78–2.08)	2.14	(1.16–3.96)
**Outcome**	**Dose Change**	**OR**	**(95%CI)**	**OR**	**(95%CI)**	**OR**	**(95%CI)**	**OR**	**(95%CI)**
More Frequent Non-Prescribed Opioid Pain Reliever Use	No Change	Reference		Reference		Reference		Reference	
	Increase	0.96	(0.84–1.11)	1.03	(0.89–1.18)	0.74	(0.55–1.00)	1.10	(0.53–2.30)
	Decrease	1.14	(0.94–1.38)	1.32	(1.08–1.61)	0.68	(0.45–1.04)	0.99	(0.36–2.75)
	Discontinued	1.75	(1.45–2.11)	1.26	(1.04–1.53)	3.94	(2.70–5.77)	2.52	(1.42–4.47)

*n = 56,484 nested cohort observations for heroin outcome; n = 56,372 for non-prescribed opioid pain reliever model

Results were similar for extended CRMs with variable odds ratios, although the association of increased OPR dose with subsequent heroin use frequency appeared more pronounced among participants reporting frequent heroin use. An additional association was found between decreased OPR dose and any non-prescribed OPR use (AOR: 1.32, 1.08–1.61). Results were also largely consistent across the two sensitivity analyses with different covariate lookback periods. (**S2 Table** and **S3 Table**).

In the models that included interaction terms between prescribed opioid dose change and illicit opioid use prior to baseline, there was no evidence of interaction effects (*p* = 0.83 for the heroin outcome model; *p* = 0.51). Results of the stratified models are presented in the supplemental materials (**S4 Table**) and estimates were largely consistent with the main CRM models. The results of the models that included benzodiazepine prescription as a lagged covariate are presented in **S5 Table**; effect estimates were qualitatively unchanged by the inclusion of this additional covariate.

## Discussion

Patients who were discontinued from prescribed OPRs were more likely to use illicit OPRs and heroin more frequently in subsequent quarters than patients who experienced no change in prescribed OPR dose. We also identified more frequent heroin use among participants who underwent an increase in OPR dose, suggesting that there were risks to both increasing and discontinuing OPR therapy.

### Strengths and limitations of the study

Our study design has both strengths and weaknesses. We conducted a blinded comparison of subjective (self-reported illicit substance use) and objective (chart prescription records) data, overcoming potential recall bias. Nonetheless, our comparisons of changes in OPR prescribing in one quarter and illicit substance use in subsequent quarters assumed accurate timing of recall in both self-report and medical charting that may have obscured actual associations. In addition, restrictions on access to California CSMP data and the limits of medical chart data restricted our ability to confirm dispensation or consumption of prescribed OPRs. While the historical reconstruction interview we utilized has strong validity and reliability, we adapted it for use in this study. Furthermore, our sample was limited to patients who were alive, able to be located, and willing to come to a visit; we may have missed the most vulnerable patients who were no longer living or were lost to follow up, and thus may have underestimated the impact of prescribing changes on patient care. Finally, results are observational and may not generalize beyond safety-net settings or settings without high levels of lifetime illicit substance use.

### Findings in context

Our results are consistent with recent literature, including a model suggesting that interventions intended to improve OPR prescribing could at least temporarily increase opioid-related harms due to increased use of illicit opioids. [39] Moreover, an analysis of Medicaid beneficiaries in Vermont found that the rapidity of OPR discontinuation was associated with a higher likelihood of subsequent opioid-related emergency department visits and hospitalizations. [40] Thus, our results support the CDC’s recent warning to exercise caution when reducing and discontinuing OPRs, and to avoid when possible unilaterally or arbitrarily discontinuing OPRs outside of a collaborative care decision. [41] Although a patient-centered plan to reduce reliance upon OPRs may result in reduced pain and improved function and quality of life, [42] achieving such a goal in clinical practice can be challenging. Additional interventions such as training and education on tapering, multi-modal pain treatment and behavioral health therapy, and increased access to medications to treat OUD may help to manage at-risk patients. In fact, of the 237 patients in our sample who experienced at least one opioid discontinuation during follow-up, only five reported initiating medications for OUD treatment in the first year following discontinuation.

The association of increased OPR dose with more frequent heroin use among those who used heroin at least intermittently, as shown in **Fig 2**, is concordant with CDC Guideline recommendations to exercise caution when increasing OPR dose. Our data did not permit determination of the reason for this finding, which may have been due to worsening pain conditions resulting in supplemental heroin use, increasing tolerance to opioids, or exchange of prescribed OPRs for heroin. We did not identify an association between reduced OPR dose and more frequent heroin use, yet there was an association between reduced OPR dose and more frequent non-prescription OPR use that was inconsistent across conditional comparisons in the extended CRM.

## Conclusions

Loss of access to prescribed OPRs was associated with more frequent use of non-prescribed opioids and heroin, and increased OPR dose was associated with more frequent heroin use. In addition to being cautious with increasing OPR dose, health care providers should consider the potential unintended consequences of stopping OPR therapy when developing opioid prescribing guidelines and managing practice.

## Supporting information

S1 Table Measure definitions and data sources. (DOCX)

S2 Table Single quarter covariate lags: Multivariable continuation ratio regression results assessing the association between changes in prescribed opioid dose and use frequency of heroin and non-prescribed opioid pain relievers (n = 56,484 nested cohort observations for heroin outcome; n = 56,372 for non-prescribed opioid pain reliever model). (DOCX)

S3 Table Four quarter covariate lags: Multivariable continuation ratio regression results assessing the association between changes in prescribed opioid dose and use frequency of heroin and non-prescribed opioid pain relievers (n = 56,484 nested cohort observations for heroin outcome; n = 56,372 for non-prescribed opioid pain reliever model). (DOCX)

S4 Table Multivariable continuation ratio regression assessing the association between changes in prescribed opioid dose and use frequency of heroin and non-prescribed opioid pain relievers, stratified by heroin use prior to baseline (for heroin outcome model) and non-prescribed opioid pain reliever use (for non-prescribed opioid pain reliever use model). (DOCX)

S5 Table Multivariable continuation ratio regression assessing the association between changes in prescribed opioid dose and use frequency of heroin and non-prescribed opioid pain relievers, including benzodiazepine prescription as a lagged covariate. (DOCX)
