# Supplementary material for: Illicit opioid use following changes in opioids prescribed for chronic non-cancer pain
Source: PLoS One. 2020 May 4;15(5):e0232538. doi: 10.1371/journal.pone.0232538 (PMC7197848; doi:10.1371/journal.pone.0232538)
Supplement: S1 Table — (DOCX) [file pone.0232538.s001.docx]

| **S1 Table**: Measure definitions and data sources | | |
| --- | --- | --- |
| **Variable Type** | **Variable** | **Source** |
| Exposure | Dose change in current quarter (No Change, Increase, Decrease, Discontinued) | Medical chart abstraction |
| Outcomes | Frequency of heroin use (none, once/intermittent, ≤weekly to few times a week, daily/nearly daily) | Historical reconstruction interview |
|  | Frequency of non-prescribed opioid pain reliever use (none, once/intermittent, ≤weekly to few times a week, daily/nearly daily) | Historical reconstruction interview |
| Covariates | Age | Computer assisted personal interview (CAPI) |
|  | Race | Computer assisted personal interview (CAPI) |
|  | Gender | Computer assisted personal interview (CAPI) |
|  | Education | Computer assisted personal interview (CAPI) |
|  | Frequency of heroin use in prior quarter(s) (none, once/intermittent, ≤weekly to few times a week, daily/nearly daily) | Historical reconstruction interview |
|  | Frequency of non-prescribed opioid pain reliever use in prior quarter(s) (none, once/intermittent, ≤weekly to few times a week, daily/nearly daily) | Historical reconstruction interview |
|  | Starting dose in current quarter | Medical chart abstraction |
|  | Dose change in prior quarter | Medical chart abstraction |
|  | Mean Opioid Dose in year prior to start of follow-up | Medical chart abstraction |
|  | Maximum alcohol use frequency in prior quarter(s) | Historical reconstruction interview |
|  | Any cocaine or meth use in prior quarter(s) | Historical reconstruction interview |
|  | Any opioid-related ED visits in prior quarter(s) | Medical chart abstraction |
|  | Any yellow flag behavior noted in chart in prior quarter(s) | Medical chart abstraction |
|  | Any pain agreement indicated as signed in prior quarter(s) | Medical chart abstraction |
|  | Any controlled substance monitoring program (CSMP) check indicated in prior quarter(s) | Medical chart abstraction |
|  | First naloxone prescription in prior quarter(s) | Medical chart abstraction |
|  | Any self-reported medication-assisted treatment in prior quarter(s) | Historical reconstruction interview |
