# Supplementary material for: Illicit opioid use following changes in opioids prescribed for chronic non-cancer pain
Source: PLoS One. 2020 May 4;15(5):e0232538. doi: 10.1371/journal.pone.0232538 (PMC7197848; doi:10.1371/journal.pone.0232538)
Supplement: S2 Table — (DOCX) [file pone.0232538.s002.docx]

| **S2 Table:** Single Quarter Covariate Lags: Multivariable continuation ratio regression results assessing the association between changes in prescribed opioid dose and use frequency of heroin and non-prescribed opioid pain relievers (n=56,484 nested cohort observations for heroin outcome; n=56,372 for non-prescribed opioid pain reliever model) | | | | | | | | | | | |
| --- | --- | --- | --- | --- | --- | --- | --- | --- | --- | --- | --- |
|  |  | **Continuation Ratio Model with Constant Odds Ratios** | | **Continuation Ratio Model with Variable Odds Ratio** | | | | | | | |
|  |  |  |  | **Any vs. None** | |  | **Weekly/Daily vs. Intermittently** | |  | **Daily vs. Weekly** | |
| **Outcome** | **Dose Change** | **OR** | **(95%CI)** | **OR** | **(95%CI)** |  | **OR** | **(95%CI)** |  | **OR** | **(95%CI)** |
| Heroin Use | No Change | Reference | | Reference | |  | Reference | |  | Reference | |
|  | Increase | 1.84 | (1.44-2.35) | 1.30 | (1.05-1.60) |  | 4.36 | (2.07-9.19) |  | 9.89 | (3.50-27.97) |
|  | Decrease | 0.86 | (0.67-1.10) | 0.99 | (0.76-1.28) |  | 0.67 | (0.37-1.23) |  | 0.56 | (0.26-1.21) |
|  | Discontinued | 1.55 | (1.23-1.96) | 1.51 | (1.19-1.92) |  | 1.24 | (0.76-2.02) |  | 2.45 | (1.31-4.57) |
| **Outcome** | **Dose Change** | **OR** | **(95%CI)** | **OR** | **(95%CI)** |  | **OR** | **(95%CI)** |  | **OR** | **(95%CI)** |
| Non-Prescribed Opioid Pain Reliever Use | No Change | Reference | | Reference | |  | Reference | |  | Reference | |
|  | Increase | 0.97 | (0.84-1.12) | 1.02 | (0.89-1.18) |  | 0.76 | (0.56-1.03) |  | 1.08 | (0.50-2.33) |
|  | Decrease | 1.11 | (0.92-1.34) | 1.28 | (1.05-1.56) |  | 0.64 | (0.42-0.98) |  | 1.11 | (0.39-3.16) |
|  | Discontinued | 1.78 | (1.47-2.15) | 1.29 | (1.07-1.56) |  | 4.05 | (2.74-5.98) |  | 2.56 | (1.43-4.57) |
