# Supplementary material for: Illicit opioid use following changes in opioids prescribed for chronic non-cancer pain
Source: PLoS One. 2020 May 4;15(5):e0232538. doi: 10.1371/journal.pone.0232538 (PMC7197848; doi:10.1371/journal.pone.0232538)
Supplement: S3 Table — (DOCX) [file pone.0232538.s003.docx]

| **S3 Table:** Four Quarter Covariate Lags: Multivariable continuation ratio regression results assessing the association between changes in prescribed opioid dose and use frequency of heroin and non-prescribed opioid pain relievers (n=56,484 nested cohort observations for heroin outcome; n=56,372 for non-prescribed opioid pain reliever model) | | | | | | | | | | | |
| --- | --- | --- | --- | --- | --- | --- | --- | --- | --- | --- | --- |
|  |  | **Continuation Ratio Model with Constant Odds Ratios** | | **Continuation Ratio Model with Variable Odds Ratio** | | | | | | | |
|  |  |  |  | **Any vs. None** | |  | **Weekly/Daily vs. Intermittently** | |  | **Daily vs. Weekly** | |
| **Outcome** | **Dose Change** | **OR** | **(95%CI)** | **OR** | **(95%CI)** |  | **OR** | **(95%CI)** |  | **OR** | **(95%CI)** |
| Heroin Use | No Change | Reference | | Reference | |  | Reference | |  | Reference | |
|  | Increase | 1.60 | (1.25-2.04) | 1.19 | (0.96-1.48) |  | 2.82 | (1.36-5.83) |  | 4.88 | (2.18-10.93) |
|  | Decrease | 0.95 | (0.75-1.21) | 1.06 | (0.82-1.37) |  | 0.71 | (0.40-1.24) |  | 0.78 | (0.35-1.72) |
|  | Discontinued | 1.65 | (1.30-2.09) | 1.62 | (1.27-2.05) |  | 1.34 | (0.82-2.21) |  | 2.35 | (1.29-4.30) |
| **Outcome** | **Dose Change** | **OR** | **(95%CI)** | **OR** | **(95%CI)** |  | **OR** | **(95%CI)** |  | **OR** | **(95%CI)** |
| Non-Prescribed Opioid Pain Reliever Use | No Change | Reference | | Reference | |  | Reference | |  | Reference | |
|  | Increase | 1.02 | (0.89-1.17) | 1.08 | (0.94-1.25) |  | 0.75 | (0.56-1.02) |  | 1.29 | (0.61-2.71) |
|  | Decrease | 1.10 | (0.90-1.34) | 1.19 | (0.97-1.47) |  | 0.77 | (0.50-1.19) |  | 1.19 | (0.40-3.58) |
|  | Discontinued | 1.78 | (1.50-2.12) | 1.33 | (1.11-1.61) |  | 3.70 | (2.65-5.17) |  | 2.44 | (1.45-4.09) |
