# Supplementary material for: Illicit opioid use following changes in opioids prescribed for chronic non-cancer pain
Source: PLoS One. 2020 May 4;15(5):e0232538. doi: 10.1371/journal.pone.0232538 (PMC7197848; doi:10.1371/journal.pone.0232538)
Supplement: S4 Table — (DOCX) [file pone.0232538.s004.docx]

| **S4 Table:** Multivariable continuation ratio regression assessing the association between changes in prescribed opioid dose and use frequency of heroin and non-prescribed opioid pain relievers, stratified by heroin use prior to baseline (for heroin outcome model) and non-prescribed opioid pain reliever use (for non-prescribed opioid pain reliever use model). | | | | | |
| --- | --- | --- | --- | --- | --- |
|  |  | **Models Stratified by Past Use*** | | | |
|  |  | **No Past Use** | | **Past Use** | |
| **Outcome** | **Dose Change** | **OR** | **(95%CI)** | **OR** | **(95%CI)** |
| Heroin Use | No Change | Reference | | Reference | |
|  | Increase | 9.13 | (3.29-25.34) | 1.62 | (1.25-2.09) |
|  | Decrease | 0.95 | (0.09-9.68) | 0.92 | (0.74-1.14) |
|  | Discontinued | 3.34 | (1.07-10.38) | 1.63 | (1.29-2.04) |
| **Outcome** | **Dose Change** | **OR** | **(95%CI)** | **OR** | **(95%CI)** |
| Non-Prescribed Opioid Pain Reliever Use | No Change | Reference | | Reference | |
|  | Increase | 1.06 | (0.73-1.55) | 0.92 | (0.79-1.07) |
|  | Decrease | 2.18 | (1.42-3.35) | 1.07 | (0.87-1.31) |
|  | Discontinued | 1.58 | (0.95-2.65) | 1.80 | (1.46-2.20) |
| *n=36,363 nested cohort observations for heroin model among 374 participants with no past use; n=20,121 for heroin model among 224 participants with past use; n=35,978 nested cohort observations for non-prescribed opioid pain reliever model among 357 participants with no past use; n=20,394 for non-prescribed opioid pain reliever model among 240 participants with past use. | | | | | |
