# Supplementary material for: Illicit opioid use following changes in opioids prescribed for chronic non-cancer pain
Source: PLoS One. 2020 May 4;15(5):e0232538. doi: 10.1371/journal.pone.0232538 (PMC7197848; doi:10.1371/journal.pone.0232538)
Supplement: S5 Table — (DOCX) [file pone.0232538.s005.docx]

| **S5 Table:**  Multivariable continuation ratio regression assessing the association between changes in prescribed opioid dose and use frequency of heroin and non-prescribed opioid pain relievers, including benzodiazepine prescription as a lagged covariate | | | | | | | | | | | |
| --- | --- | --- | --- | --- | --- | --- | --- | --- | --- | --- | --- |
|  |  | **Continuation Ratio Model with Constant Odds Ratios** | | **Continuation Ratio Model with Variable Odds Ratio** | | | | | | | |
|  |  |  |  | **Any vs. None** | |  | **Weekly/Daily vs. Intermittently** | |  | **Daily vs. Weekly** | |
| **Outcome** | **Dose Change** | **OR** | **(95%CI)** | **OR** | **(95%CI)** |  | **OR** | **(95%CI)** |  | **OR** | **(95%CI)** |
| Heroin Use | No Change | Reference | | Reference | |  | Reference | |  | Reference | |
|  | Increase | 1.70 | (1.34-2.15) | 1.21 | (0.99-1.49) |  | 4.08 | (1.91-8.71) |  | 6.60 | (2.54-17.13) |
|  | Decrease | 0.86 | (0.67-1.11) | 0.99 | (0.77-1.28) |  | 0.64 | (0.35-1.16) |  | 0.60 | (0.27-1.33) |
|  | Discontinued | 1.56 | (1.25-1.94) | 1.58 | (1.27-1.97) |  | 1.17 | (0.73-1.90) |  | 2.13 | (1.15-3.96) |
| **Outcome** | **Dose Change** | **OR** | **(95%CI)** | **OR** | **(95%CI)** |  | **OR** | **(95%CI)** |  | **OR** | **(95%CI)** |
| Non-Prescribed Opioid Pain Reliever Use | No Change | Reference | | Reference | |  | Reference | |  | Reference | |
|  | Increase | 0.95 | (0.83-1.09) | 1.02 | (0.89-1.18) |  | 0.72 | (0.53-0.97) |  | 1.00 | (0.47-2.10) |
|  | Decrease | 1.14 | (0.94-1.38) | 1.32 | (1.07-1.61) |  | 0.69 | (0.46-1.06) |  | 0.89 | (0.34-2.35) |
|  | Discontinued | 1.73 | (1.44-2.09) | 1.25 | (1.03-1.52) |  | 3.88 | (2.68-5.63) |  | 2.60 | (1.48-4.58) |
| *n=56,484 nested cohort observations for heroin outcome; n=56,372 for non-prescribed opioid pain reliever model | | | | | | | | | | | |
